# Elevated urinary N-acetyl-β-D-glucosaminidase is associated with high glycoalbumin-to-hemoglobin A1c ratio in type 1 diabetes patients with early diabetic kidney disease

**DOI:** 10.1038/s41598-018-25023-5

**Published:** 2018-04-30

**Authors:** Namki Hong, Minyoung Lee, Soyoung Park, Yong-ho Lee, Sang-Man Jin, Jae Hyeon Kim, Byung-Wan Lee

**Affiliations:** 1 Division of Endocrinology and Metabolism, Department of Internal Medicine, Yonsei University College of Medicine, Seoul, Korea; 2 Division of Endocrinology and Metabolism, Department of Medicine, Samsung Medical Center, Sungkyunkwan University School of Medicine, Seoul, Korea

## Abstract

Urinary N-acetyl-β-D-glucosaminidase (uNAG) predicted the progression of diabetic kidney disease (DKD) prior to development of albuminuria in diabetes patients. We sought whether uNAG level is associated with glycoalbumin-to-hemoglobin A1c ratio (G/A ratio), a marker of postprandial hyperglycemia and glycemic excursion, independent of albuminuria and kidney function. The association between uNAG excretion and G/A ratio was assessed in 204 consecutive subjects with type 1 diabetes (T1D) (mean age 43.9 years; 49.0% men). uNAG excretion level increased along with older age, hyperglycemia, and degree of albuminuria, but was not correlated with body mass index or estimated glomerular filtration rate (eGFR). Elevated uNAG showed robust association with higher G/A ratio (adjusted β = 0.103, P = 0.020) after adjustment for age, sex, body mass index, duration of diabetes, uACR, angiotensin blockers use, fasting plasma glucose, and hemoglobin level. uNAG showed better discriminatory performance for individuals with high G/A ratio than albuminuria (AUC 0.613 vs. 0.518, P = 0.038). Measurement of uNAG improved AUC for high G/A ratio from 0.699 to 0.748 (P = 0.043) when added to conventional risk factors (cutoff 5.24 U/g creatinine; sensitivity 62.5% and specificity 58.0%). In conclusion, Elevated uNAG was found to be associated with high G/A ratio in patients with T1D with early stage DKD, independent of age and albuminuria.

## Introduction

The health and economic burden of diabetic kidney disease (DKD) has increased significantly due to its strong association with cardiovascular disease and end-stage renal disease^1^. Given the reduced risk of albuminuria and DKD progression by intensive treatment in early stage DKD demonstrated in previous large trials, the early identification and monitoring of DKD is critical, particularly in type 1 diabetes (T1D)^2^. In current clinical practice, identification of DKD depends on the assessment of kidney function using estimated glomerular filtration rate (eGFR) derived from formulas based on serum creatinine values and kidney damage as reflected by albuminuria^1^. However, eGFR and albuminuria have some limitations in estimating the risk of DKD progression in the early stages of DKD. eGFR was shown to be less precise in the higher GFR range due to the potential for overestimation, also referred to as hyperfiltration^3^. Although albuminuria, usually defined as 30 mg/g creatinine or more, is often the first clinical indicator of DKD, prior studies revealed that the relationship of albuminuria to end-stage renal disease and cardiovascular disease began even at ‘normal’ levels under the 30 mg/g creatinine cutoff as a continuum^4^. Thus, a more sensitive marker that can reflect subclinical kidney damage due to glucose dysregulation is needed to improve the risk stratification and monitoring of DKD in T1D patients in the early stages of DKD.

Urinary N-acetyl-β-D-glucosaminidase (uNAG), a marker of renal proximal tubule injury, is known to increase with hyperglycemia, even in normoalbuminuric conditions, and to decrease with improved glycemic control in patients with diabetes^5,6^. In a nested case-control study of T1D patients with early DKD in the Diabetes Control and Complications Trial (DCCT), uNAG and submicroalbuminuric albumin excretion rate predicted the onset of micro- and macroalbuminuria, independent of each other^7^. The association between uNAG and DKD progression was shown to be independent of hyperglycemia assessed by hemoglobin A1c (A1c) level, indicating that uNAG excretion might reflect the hyperglycemia-independent component of glycemic dysregulation^7^. Glycoalbumin (GA)-to-A1c ratio (G/A) ratio has been proposed as a sensitive marker reflecting postprandial hyperglycemia, glycemic excursion, and decreased beta cell function capacity in patients with diabetes^8–14^. Postprandial hyperglycemia and glycemic excursion has been postulated as a distinctive, crucial pathogenesis of micro- and macrovascular complications^15^. However, it remains unclear whether elevated uNAG level is associated with high G/A ratio among T1D patients with early stage DKD.

In this study, we aimed to investigate whether uNAG excretion level is positively associated with G/A ratio, independent of albuminuria. We further investigated whether the measurement of uNAG might have additive discriminatory value for the identification of T1D patients at risk for progression of DKD, in addition to conventional risk factors such as hyperglycemia and albuminuria.

## Materials and Methods

### Study participants and design

In this retrospective cross-sectional study, the study population consisted of consecutive T1D patients who visited the diabetes clinic of a tertiary-level institution (Samsung Medical Center and Severance Hospital, Seoul, Korea) between March 2015 and November 2016. T1D was diagnosed based on clinical characteristics, positivity for anti-glutamic acid decarboxylase antibody, or a decreased C-peptide level below 0.8 ng/mL at the initial visit. Among 246 subjects, individuals without uNAG data (N = 5), without GA measurement (N = 12), and those being treated with sodium-glucose co-transporter 2 inhibitors (N = 7) were excluded (Supplementary Fig. 1). Early DKD was defined in accordance with KDIGO guidelines as follows:^4^ subjects with normal or mildly decreased GFR (G1 or G2; eGFR ≥ 60 mL/min/1.73 m^2^) and albuminuria equal to or less than a moderately increased level (A1 or A2; < 300 mg/g creatinine). Thus, subjects with eGFR < 60 mL/min/1.73 m^2^ (N = 13) or severe albuminuria (N = 11; ≥ 300 mg/g creatinine) were further excluded from analysis. For the remaining 204 subjects, data regarding demographics, body mass index (BMI), comorbidities, and medication use were collected at a single time point defined as the first visit during the study period. None of the conditions that might alter albumin or hemoglobin metabolism that could affect A1c and GA values including malignancy, liver cirrhosis, hematologic diseases, thyroid dysfunction, regular steroid use, or pregnancy were found in any of the subjects after an individual-level medical record review. This study was approved by the institutional review board of Samsung Medical Center and Severance Hospital (IRB No. 4-2015-0828), which waived written informed consent due to the retrospective nature of the study.

### Assessment of glucometabolic parameters and kidney function

Fasting blood samples were obtained to measure the glucometabolic parameters. A1c level was measured by high-performance liquid chromatography using Variant II Turbo (Bio-Rad Laboratories; Hercules, CA, USA; % CV of intra-assay variability <1.3%). Serum GA level was determined by enzymatic method using an albumin-specific proteinase, ketoamine oxidase, and an albumin assay reagent (LUCICA GA-L assay kit, Asahi Kasei Pharma Co, Tokyo, Japan; % CV <3%) using the Hitachi 7699 P module autoanalyzer (Hitachi Instruments Service, Tokyo, Japan). The reference ranges of A1c and GA were between 4.0% and 6.0% and between 11.0% and 16.0%, respectively. G/A ratio was analyzed as a continuous variable and also as a categorical variable using the cutoff value of 2.8 for high G/A ratio^9^. Serum C-peptide level was assessed using an immunoradiometric assay method (Beckman Coulter, Fullerton, CA, USA). Serum concentration of fasting glucose and total cholesterol were measured by standard methods. Serum creatinine levels were determined with a Hitachi 7600-110 automated chemistry analyzer (Hitachi Co., Tokyo, Japan) with CREA (Roche Diagnostics, Indianapolis, IN, USA) standardized to isotope dilution mass spectrometry. As recent study showed that the original Chronic Kidney Disease Epidemiology Collaboration (CKD-EPI) equation and Korean version equation had equivalent overall analytic performance, eGFR was calculated using the original CKD-EPI equation based on serum creatinine level^16,17^.

### Urinary markers for glomerular and tubular damage

Fasting morning urine sample was used to measure urinary albumin, NAG, and creatinine excretion. Albuminuria and NAG excretion was standardized to urinary creatinine excretion. Urinary albumin level was measured by an immunoturbidimetric method and urine creatinine by a kinetic Jaffe method using an AU680 automated chemistry analyzer (Beckman Coulter, CA, USA). Albuminuria was analyzed as a continuous variable or a categorized variable according to KDIGO guidelines (normal to mildly increased albuminuria [A1], albumin-to-creatinine ratio (ACR) less than 30 mg/g creatinine, n = 176; moderate albuminuria [A2], ACR between 30 and 300 mg/g creatinine, n = 28)^4^. uNAG was measured by an enzymatic method (Nittobo Medical, Fukushima, Japan) using a JCA-BM 6010/c automated chemistry analyzer (JEOL Ltd, Tokyo, Japan). A high uNAG level was defined using 5.8 U/g creatinine as a dichotomized cutoff, based on the upper normal value in a study of the Japanese population (normal range: 1.6 U/g to 5.8 U/g creatinine)^18^.

### Statistical analysis

Data are presented as mean ± SD, median (interquartile range [IQR]), or numbers (percentages). Baseline characteristics of subjects were compared using student’s t test, Wilcoxon rank-sum test, or Pearson’s chi-square test as appropriate. Median uNAG level or mean log-uNAG level among groups combination of G/A ratio and albuminuria were compared using the Kruskal-Wallis test with Dunn’s procedure or analysis of covariance (ANCOVA) with adjustment for age and duration of diabetes. Due to the non-normality distribution of the uNAG level that exhibited a right-skewed pattern (Kolmogorov-Smirnov P < 0.01), natural log-transformed uNAG (log-uNAG, Kolmogorov-Smirnov P = 0.963) was used in the correlation analyses and regression models. The correlation between log-uNAG, glucometabolic parameters, and log-transformed uACR (log-uACR) was assessed by Pearson’s correlation coefficient. Multivariate linear regression models were built to elucidate the independent association of uNAG with the G/A ratio after adjustment for covariates including age, sex, BMI, duration of diabetes, log-uACR, angiotensin blockers use, fasting plasma glucose, and hemoglobin level. For sensitivity analysis, uNAG was entered into multivariate linear regression model as either a continuous variable (log-uNAG) or a categorical variable (uNAG quintiles). Area under the receiver operating characteristic curve (AUC) was compared to evaluate whether uNAG measurement could further improve the identification of individuals with high G/A ratio, in addition to conventional risk factors including uACR, with calculation of the optimal cutoff using Youden’s index^19^. We calculated category-free net reclassification improvement (NRI), absolute integrated discrimination improvement (IDI), and relative IDI described by Pencina *et al*., with the help of STATA add-on^20^. Two-sided P values less than 0.05 were considered statistically significant. All statistical analyses were performed using STATA 14.0 (StataCorp, College Station, TX, USA).

### Data availability

The datasets used and/or analyzed during this study are available from the corresponding author on reasonable request.

## Results

### Baseline characteristics of the study participants

The mean age of the study participants was 43.9 ± 14.9 years and 100 (49.0%) were men (Table 1). The median uNAG level was 5.6 U/g creatinine with an interquartile range of 3.2–9.9. Compared with subjects with uNAG levels within the predefined normal range (N = 105, 51.5%), those with high uNAG levels were older in age and had higher GA, A1c, and ACR levels. The High uNAG group had longer diabetes duration and marginally higher fasting plasma glucose levels, whereas BMI did not differ significantly between two groups.

**Table 1:** Baseline characteristics of the study subjects by uNAG excretion level.

	Total (N = 204)	uNAG ≥ 5.80 U/g creatinine N = 99 (48.5%)	uNAG < 5.80 U/g creatinine N = 105 (51.5%)	P value
Demographics				
Age, year	**43**.**9 ± 14**.**9**	**48**.**9 ± 14**.**7**	**39**.**3 ± 13**.**6**	**<0**.**001**
Male sex	100 (49.0)	42 (42.4)	58 (55.2)	0.067
Diabetes duration, year	**11**.**7** **±** **8**.**8**	**13**.**0** **±** **9**.**8**	**10**.**5** **±** **7**.**7**	**0**.**049**
BMI, kg/m^2^	22.9 ± 3.2	23.1 ± 2.9	22.7 ± 3.5	0.422
SBP, mmHg	121.3 ± 17.4	120.6 ± 17.0	122.0 ± 16.1	0.654
Hemoglobin, g/dL	13.8 ± 1.7	13.6 ± 1.7	14.0 ± 1.6	0.104
Glucometabolic parameters				
FPG, mg/dL	162.8 ± 69.2	171.5 ± 73.7	154.5 ± 64.0	0.080
Glycoalbumin, %	**23**.**4** ± **6**.**5**	**25**.**5** ± **7**.**2**	**21**.**4** ± **5**.**1**	**<** **0**.**001**
A1c, % (mmol/mol)	**7**.**7 (61) ± 1**.**4**	**8**.**3 (67) ± 1**.**5**	**7**.**2 (55) ± 1**.**2**	**<** **0**.**001**
Fasting C-peptide, ng/mL	**0**.**13 [0**.**02–0**.**54]**	**0**.**09 [0**.**02–0**.**48]**	**0**.**20 [0**.**04–0**.**66]**	**0**.**036**
Cholesterol, mg/dL	170.9 ± 32.9	173.0 ± 38.0	168.8 ± 27.3	0.365
DN markers				
eGFR, ml/min/1.73 m^2^	**101**.**4 ± 18**.**0**	**97**.**7 ± 19**.**1**	**104**.**9 ± 16**.**2**	**0**.**004**
uACR, mg/g	**8**.**8 [5**.**5–16**.**2]**	**13**.**0 [8**.**0–26**.**4]**	**6**.**1 [4**.**1–10**.**1]**	**<0**.**001**
uNAG, U/g creatinine	**5**.**6 [3**.**2–9**.**9]**	**10**.**3 [7**.**7–16**.**8]**	**3**.**3 [2**.**2–4**.**6]**	**<0**.**001**

Data are presented as mean ± standard deviation, median [interquartile range], or as numbers (%). Bold characters represent statistically significant values. Abbreviations: A1c, hemoglobin A1c; BMI, body mass index; SBP, systolic blood pressure; FPG, fasting plasma glucose; DN, diabetic nephropathy; uNAG, urinary N-acetyl-β-D-glucosaminidase to creatinine ratio; eGFR, estimated glomerular filtration rate; uACR, urinary albumin-to-creatinine ratio.

### Correlation between urinary markers and glucometabolic parameters

Log-uNAG showed a modest positive correlation with log-uACR (r = 0.422, P < 0.001; Table 2). As expected, both log-uNAG and log-uACR were positively correlated with HbA1c (r = 0.339 and r = 0.252, respectively) and GA (r = 0.321 and r = 0.212, respectively), but eGFR (r = −0.166 and r = −0.142, respectively) showed a negatively weak correlation with both urinary markers, with significance. A distinctive pattern was observed between log-uNAG and log-uACR in terms of age and diabetes duration: elevated log-uNAG was more closely associated with older age, whereas log-uACR was positively correlated with longer duration of diabetes. BMI and systolic blood pressure did not show a significant linear correlation with either log-uNAG or log-uACR.

**Table 2:** Correlation between urinary markers and glucometabolic parameters.

	Log-uNAG	P	Log-uACR	P
	r		r	
Age, year	**0**.**319**	**<0**.**001**	0.109	0.117
BMI, kg/m^2^	0.011	0.874	−0.033	0.643
Diabetes duration, year	0.087	0.214	**0**.**162**	**0**.**019**
SBP, mmHg	0.032	0.736	0.149	0.117
Hemoglobin, g/dL	−0.096	0.170	−0.078	0.265
FPG, mg/dL	0.079	0.261	**0**.**141**	**0**.**043**
Glycated albumin, %	**0**.**321**	**<0**.**001**	**0**.**212**	**0**.**002**
A1c, %	**0**.**339**	**<0**.**001**	**0**.**252**	**0**.**003**
eGFR, mL/min/1.73 m^2^	**−0**.**166**	**0**.**017**	**−0**.**142**	**0**.**042**
uNAG, log unit*	—	—	**0**.**422**	**<0**.**001**
uACR, log unit*	**0**.**422**	**<0**.**001**	—	—

^*^uNAG and uACR were log-transformed to achieve normal distribution for calculating Pearson correlation coefficients. Bold characters represent statistically significant values. Abbreviations: A1c, hemoglobin A1c; BMI, body mass index; SBP, systolic blood pressure; FPG, fasting plasma glucose; uNAG, urinary N-acetyl-β-D-glucosaminidase; eGFR, estimated glomerular filtration rate; uACR, urinary albumin-to-creatinine ratio.

### Determinants of uNAG excretion

Independent determinants of log-uNAG level were assessed using multivariate regression models in T1D subjects (Table 3). In model 2, higher age, log-uACR, and female sex were significantly associated with elevated NAG excretion. When GA or A1c was entered into the model separately (models 3 and 4), age was the strongest determinant of log-uNAG level, followed by GA or A1c, independent of log-uACR level. Log-uNAG level did not differ significantly according to BMI, duration of diabetes, or eGFR in this study.

**Table 3:** Determinants of uNAG excretion level in subjects with type 1 diabetes.

Variables	Model 1		Model 2		Model 3		Model 4	
	st β*	P	st β	P	st β	P	st β	P
Age	**0**.**331**	**<0**.**001**	**0**.**302**	**<0**.**001**	**0**.**312**	**<0**.**001**	**0**.**309**	**<0**.**001**
Women vs. men	**0**.**191**	**0**.**006**	**0**.**126**	**0**.**050**	**0**.**133**	**0**.**029**	**0**.**141**	**0**.**021**
Diabetes duration	−0.018	0.800	−0.066	0.322	−0.053	0.397	−0.038	0.548
BMI	−0.001	0.990	0.013	0.834	0.039	0.511	−0.001	0.975
Log-uACR			**0**.**369**	**<0**.**001**	**0**.**306**	**<** **0**.**001**	**0**.**288**	**<0**.**001**
Glycoalbumin					**0**.**282**	**<0**.**001**		
A1c							**0**.**285**	**<** **0**.**001**

^*^Standardized beta coefficient was calculated to compare the relative contribution of each variable in determining the uNAG excretion level. Covariates included in each model are as follows: Model 1: age, sex, duration of diabetes, BMI; model 2: model 1 + log-uACR; model 3: model 2 + glycoalbumin; model 4: model 2 + hemoglobin A1c. Bold characters represent statistically significant values. uNAG and uACR were log-transformed for linear regression models. Abbreviations: A1c, hemoglobin A1c; uNAG, urinary N-acetyl-β-D-glucosaminidase; BMI, body mass index; uACR, urinary albumin-to-creatinine ratio.

### Comparison of uNAG level by G/A ratio and albuminuria groups

**Figure 1:**
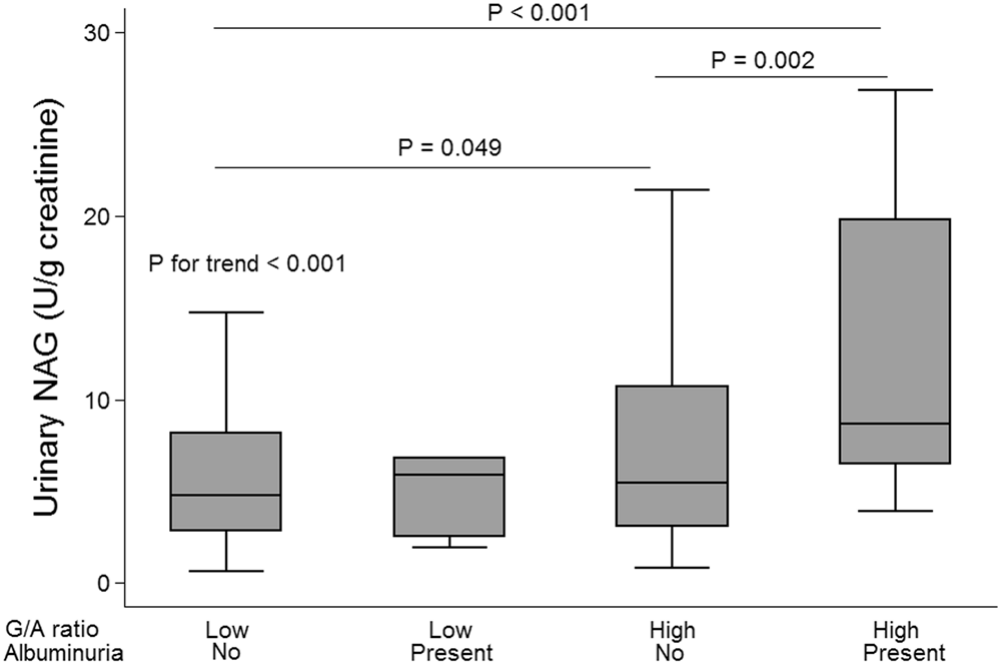
Comparison of uNAG level by combining the glycoalbumin-to-hemoglobin A1c (G/A) ratio and presence of albuminuria in subjects with type 1 diabetes. Low G/A ratios are indicated by values less than 2.8, whereas G/A ratios equal to 2.8 or more were defined as high. Albuminuria was defined as urinary albumin-to-creatinine ratio equal to or greater than 30 mg/g creatinine. P values for comparison between groups were calculated using Dunn’s procedure in order to correct for multiple comparison error. Abbreviations: uNAG, urinary N-acetyl-β-D-glucosaminidase; G/A ratio, glycoalbumin-to-hemoglobin A1c ratio.

When subjects were grouped into 4 groups according to a combination of G/A ratio (low: < 2.8 vs. high: ≥ 2.8) and albuminuria (normoalbuminuria <30 mg/g creatinine vs. microalbuminuria ≥ 30 mg/g creatinine; Fig. 1), subjects with high G/A ratios had higher median uNAG excretion levels compared to those with low G/A ratios in both the normoalbuminuria (5.5 vs. 4.8 U/g creatinine, P = 0.049) and microalbuminuria groups (8.6 vs. 5.9 U/g creatinine, P = 0.036). When mean log-uNAG levels were compared, higher mean log-uNAG level was observed in high G/A ratio group than in low G/A ratio group, even after adjustment for age and duration of diabetes (in overall: 1.9 vs 1.5 log-U/g creatinine, P = 0.004; in normoalbuminuria group: 1.8 vs 1.5 log-U/g creatinine, P = 0.047; in microalbuminuria group: 2.5 vs. 1.4 log-U/g creatinine, P = 0.012).

### Association between uNAG and G/A ratio

In T1D subjects with early DKD, elevated log-uNAG was associated with higher G/A ratios (Table 4; unadjusted β = 0.092, P = 0.015), whereas log-uACR did not show a significant association. The positive association of log-uNAG with the G/A ratio was robust (adjusted β = 0.103, P = 0.020) even after adjustment for potential covariates including age, sex, BMI, duration of diabetes, log-uACR, angiotensin blockers use, fasting plasma glucose, and hemoglobin level. When the analysis was confined to subjects with normal to mildly increased albuminuria (A1, uACR < 30 mg/g creatinine; n = 176), subjects with high uNAG (≥5.8 U/g creatinine) had higher GA (20.9 vs. 24.8%, P < 0.001), higher A1c (8.1 vs. 7.2, P < 0.001), and higher G/A ratio (3.1 vs. 2.8, P = 0.044) compared to subjects with low uNAG level (<5.8 U/g creatinine; Supplementary Fig. 2). Positive association of log-uNAG with G/A ratio remained independent (adjusted β = 0.127, P = 0.011) in subjects with A1 state (Supplementary Table 1). When uNAG was entered into the multivariate model as categorical variable (quintiles) for sensitivity analysis, we observed increasing trend of G/A ratio from lowest to higher quintiles of uNAG, where subjects with uNAG at fourth (uNAG 7.17–11.61 U/g) and fifth quintiles (uNAG ≥ 11.62 U/g) showed association with elevated G/A ratio in the multivariate model (adjusted β = 0.241, P = 0.048 and adjusted β = 0.324, P = 0.014, respectively) compared to those with lowest (first) uNAG quintile (uNAG 0.63–2.77 U/g). Subjects with second (2.78–4.70 U/g) and third uNAG quintiles (4.80–7.16 U/g) showed increasing G/A ratio in multivariate model (adjusted β = 0.097, P = 0.379 and adjusted β = 0.087, P = 0.483, respectively), although it did not reach statistical significance.

**Table 4:** Association of uNAG excretion with G/A ratio in subjects with type 1 diabetes.

Variables	Univariate	P	Multivariate	P
	Unadjusted β (95% CI)		Adjusted β (95% CI)	
uNAG, log unit	**0**.**092 (0**.**018 to 0**.**166)**	**0**.**015**	**0**.**103 (0**.**016 to 0**.**189)**	**0**.**020**
Age, year	0.001 (−0.058 to 0.060)	0.971	−0.001 (−0.005 to 0.004)	0.761
Women (vs. men)	0.089 (−0.040 to 0.220)	0.177	0.016 (−0.119 to 0.153)	0.811
BMI, kg/m^2^	**−0**.**030 (−0**.**051 to −0**.**008)**	**0**.**005**	**−0**.**028 (−0**.**049 to −0**.**007)**	**0**.**007**
Diabetes duration, year	0.105 (−0.024 to 0.234)	0.112	**0**.**173 (0**.**041 to 0**.**313)**	**0**.**011**
uACR, log unit	0.033 (−0.028 to 0.095)	0.286	−0.007 (−0.080 to 0.075)	0.952
Angiotensin blockers use (yes vs. no)	−0.121 (−0.300 to 0.058)	0.185	−0.212 (−0.417 to 0.005)	0.055
FPG, mg/dL	**0**.**014 (0**.**005 to 0**.**024)**	**0**.**002**	**0**.**016 (0**.**006 to 0**.**025)**	**0**.**002**
Hemoglobin, g/dL	−0.014 (−0.053 to 0.024)	0.471	0.006 (−0.033 to 0.045)	0.782

uNAG and uACR were log-transformed for linear regression models (log-uNAG and log-uACR). In multivariate model, β coefficient of uNAG for G/A ratio was reported with adjustment for age, sex, BMI, duration of diabetes, log-uACR, angiotensin blockers use, FPG, and hemoglobin. Bold characters represent statistically significant values. Abbreviations: uNAG, urinary N-acetyl-β-D-glucosaminidase; BMI, body mass index; uACR, urinary albumin-to-creatinine ratio.

### Additive discriminatory value of uNAG for subjects with high G/A ratio

**Figure 2:**
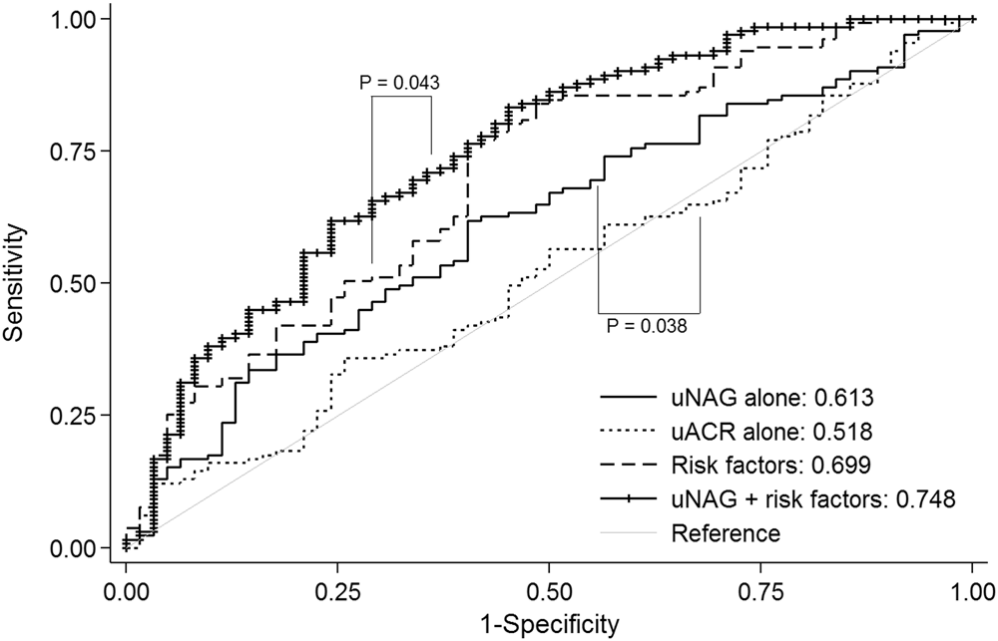
Measurement of uNAG improved the identification of subjects with higher G/A ratios, in addition to conventional risk factors such as uACR. The solid line, very short dash line, dash line, and line with small cross represent the discriminatory ability characterized by the area under the curve (AUC) for log-uNAG alone, log-uACR alone, for the model that included conventional risk factors (log-uACR, age, sex, BMI, diabetes duration, angiotensin blocker use, eGFR, and fasting plasma glucose level), and for the composite of log-uNAG and the conventional risk model, respectively. Abbreviations: log-uNAG, log-transformed urinary N-acetyl-β-D-glucosaminidase; log-uACR, log-transformed urinary albumin-to-creatinine ratio; ROC, receiver operating characteristics curve; BMI, body mass index; eGFR, estimated glomerular filtration rate; G/A ratio, glycoalbumin-to-hemoglobin A1c ratio.

The discriminatory value of uNAG for high G/A ratio was assessed by comparing the area under the curve (AUC) in a receiver operating characteristic (ROC) analysis (Fig. 2). The AUC of log-uNAG alone for discrimination of high G/A ratio was 0.613, which was significantly higher than the AUC of the log-uACR (0.613 vs. 0.518, P = 0.038). When log-uNAG level was added to the conventional risk factors including log-uACR, age, sex, BMI, diabetes duration, angiotensin blocker use, fasting plasma glucose, and hemoglobin level, its discriminatory performance significantly improved from AUC 0.699 to 0.748 (P = 0.043). The optimal cutoff of uNAG for high glycemic variability (G/A ratio ≥ 2.8) was 5.24 U/g creatinine (1.65 log unit), with a sensitivity and specificity of 62.5% and 58.0%, respectively. Measurement of log-uNAG significantly improved the identification of high glycemic variability when added to conventional model including log-uACR (category-free NRI 0.342, 95% CI 0.040–0.643, P = 0.027; absolute IDI 0.042, 95% CI 0.016–0.067, P = 0.002; relative IDI 0.357, 95% CI 0.123–0.590, P = 0.002), which was consistent with the results of the ROC analysis.

## Discussion

In this study, we found that the uNAG level was significantly elevated in subjects with T1D in the early stages of DKD with high G/A ratio compared to those with normal to low G/A ratio. The association between elevated uNAG and high G/A ratio was independent of the presence of albuminuria. uNAG showed better discriminatory performance for individuals with high G/A ratio compared to uACR. Measurement of the uNAG level in T1D significantly improved identification of subjects with high G/A ratio in addition to conventional risk factors including age, duration of diabetes, and albuminuria.

It is well known that kidney tubule damage plays an important role in the pathogenesis of DKD^21^. uNAG, a 130 kDa-sized lysosomal enzyme in the proximal tubule epithelial cells, is a well-studied specific marker of renal tubular injury^22^. Regarding the clinical relevance of uNAG in terms of conventional glucometabolic risk factors, uNAG level was known to be higher in patients with diabetes compared to healthy controls and to increase along with age, hyperglycemia, and the degree of albuminuria^5^. Even before the development of albuminuria, uNAG excretion was found to be positively correlated with hyperglycemia and diabetes duration^5^. Among our T1D subjects, the median uNAG level (5.6 U/g creatinine) fell within the reported range of values in various populations with diabetes (2.0–10.1 U/g creatinine), which was close to the range reported in a study of T1D subjects of similar age^18,23–25^. In accordance with previous findings, age was the most important determinant of uNAG level with significant linear correlation in this study, followed by poor glycemic control (A1c or GA level) and degree of albuminuria (ACR) in the submicroalbuminuric range.

In this study, we found that an elevated uNAG level was associated with high G/A ratio, independent of hyperglycemia and albuminuria, in type 1 diabetes patients in the very early stages of DKD with intact kidney function and normo- (in most cases, 86.3%) to microalbuminuria. A better discriminatory performance of uNAG for subjects with high G/A ratio was observed compared to albuminuria in our study subjects. Our finding is supported by findings from previous studies in which an elevation of uNAG excretion was reported to precede the development of albuminuria, decline of eGFR, and progression to fulminant DKD, even in prediabetes subjects^5–7,23^. These findings emphasize the potential role of renal tubular injury as a primary pathophysiology in early DKD^21,26^. Although proteinuria derived from glomerular leakage is known to induce tubulointerstitial damage, studies using urinary tubule injury markers, including uNAG, support that proximal tubule injury contributes as a priming event to the development of early DKD via hyperplasia, hyperresorption, and tubular senescence leading to glomerular hyperfiltration, interstitial inflammation, and glomerulosclerosis^21,26^. However, as suggested by the results from the DCCT, sustained hyperglycemia at fasting status alone could not fully explain the observed association between uNAG and the development of albuminuria^7^. Postprandial hyperglycemia has been noted as an important risk factor for progression of renal arteriosclerosis and tubulointerstitial lesions^27^. Monnier and colleagues reported that glucose fluctuation caused a more specific triggering effect on oxidative stress than did chronic sustained hyperglycemia in patients with diabetes^28^. G/A ratio has been proposed as a marker of postprandial hyperglycemia, glycemic excursion, and decreased insulin secretion capacity, which was also associated with the presence of DKD^8,10–14,29^. In line with these findings, we found that a urinary tubular damage marker, uNAG, was positively associated with G/A ratio, independent of albuminuria and fasting glucose level. Even when analyses were confined to subjects without albuminuria, small but discernable elevation of median uNAG level was observed in high G/A ratio group compared to normal G/A ratio group. The elevation of uNAG level was more prominent in albuminuria group, suggesting the distinctive role of uNAG reflecting early renal tubular damage in T1D patients. Furthermore, uNAG measurement improved the discrimination for subjects with high G/A ratio when added to conventional risk factors. Taken together, our findings may support the potential utility of uNAG as a sensitive renal tubular damage marker which reflects poor glycemic control status in very early stage of DKD, particularly before the onset of albuminuria, although further prospective studies are needed to validate these findings.

We acknowledge that the current study has several limitations. Due to the cross-sectional design of this study, we could not make any inference on causality. Although a nested case-control study with relatively small sample size provided evidence regarding the association of uNAG and progression of DKD, whether individuals with elevated uNAG actually progress to develop advanced DKD, independent of the albuminuria, should be confirmed in long-term, large prospective studies^7^. Although several studies suggested the potential utility of G/A ratio as a indicator of postprandial hyperglycemia, glycemic excursion, decreased beta cell capacity, and presence of DKD, whether G/A ratio actually mediates the association between uNAG with progression of DKD or onset of other vascular complications of diabetes need to be further investigated^8,10–14,29^. Blood and urine samples obtained at a single time were measured. It would be helpful to consolidate our findings by measuring other markers of renal tubule injury, which were not available in this study^7^. Koki Mise and colleagues provided evidence that interstitial fibrosis and tubular atrophy score had superior prognostic utility than uNAG for progression of diabetic nephropathy in patients with type 2 diabetes^30^. Although renal biopsy data were not available in this study, further study with tubulointerstitial pathology might strengthen our hypothesis on the association between glycemic variability and early tubulointerstitial change. Angiotensin blockers are known to decrease uNAG excretion^31^. Although information regarding the dosing and duration of angiotensin blockers could not be analyzed, we adjusted the use of angiotensin blockers at the time of measurement as a dichotomized variable in the multivariate model, which did not attenuate the association between uNAG and G/A ratio.

## Conclusions

Elevated uNAG excretion was associated with subjects with high G/A ratio among T1DM patients with early DKD, independent of age, BMI, fasting glucose level, and albuminuria. uNAG measurement improved the ability to discriminate individuals with high G/A ratio, in addition to conventional risk factors. Our findings might suggest the potential role of uNAG as an early tubular damage marker reflecting poor glycemic control in patients with type 1 diabetes at early stage of DKD, independent of albuminuria.

## Electronic supplementary material

Supplementary Information
