# Supplementary material for: Elevated urinary N-acetyl-β-D-glucosaminidase is associated with high glycoalbumin-to-hemoglobin A1c ratio in type 1 diabetes patients with early diabetic kidney disease
Source: Sci Rep. 2018 Apr 30;8:6710. doi: 10.1038/s41598-018-25023-5 (PMC5928247; doi:10.1038/s41598-018-25023-5)
Supplement: Supplementary file 1 — Supplementary Information [file 41598_2018_25023_MOESM1_ESM.pdf]

Title: Elevated urinary N-acetyl-β-D-glucosaminidase is associated with high glycoalbumin-to-hemoglobin A1c ratio in type 1 diabetes patients with early diabetic kidney disease

Namki Hong, MD, MPH[1], Minyoung Lee, MD[1], Soyoung Park, MD[2], Yong-ho Lee, MD, PhD[1], Sang-Man Jin, MD, PhD[2], Jae Hyeon Kim, MD, PhD[2], Byung-Wan Lee, MD, PhD[1]

[1]Division of Endocrinology and Metabolism, Department of Internal Medicine, Yonsei University College of Medicine, Seoul, Korea

[2]Division of Endocrinology and Metabolism, Department of Medicine, Samsung Medical Center, Sungkyunkwan University School of Medicine, Seoul, Korea

Co-Corresponding authors:

Jae Hyeon Kim, MD, PhD

Division of Endocrinology and Metabolism, Department of Medicine, Samsung Medical Center, Sungkyunkwan University School of Medicine, 81-Seoul, Irwon-ro, Gangnam-gu, Seoul 06351, Korea

Email: jaehyeon@skku.edu

Byung-Wan Lee, MD, PhD

Division of Endocrinology and Metabolism, Department of Internal Medicine, Yonsei University College of Medicine, 50-1 Yonsei-ro, Seodaemun-gu, Seoul 03722, Korea

Office number: +82-2-2228-1938

Fax number: +82-2-393-6884

Email: bwanlee@yuhs.ac

Supplementary Table 1. Association of uNAG excretion with G/A ratio in a subset of study subjects with normal to mildly increased albuminuria (A1, albumin-to-creatinine ratio less than 30 mg/g creatinine)

	Univariate		Multivariate	
Variables	β (95% CI)	P	β (95% CI)	P
uNAG, log unit	**0.090 (0.007 to 0.172)**	**0.032**	**0.127 (0.029 to 0.225)**	**0.011**
Age, year	0.014 (-0.051 to 0.079)	0.668	0.001 (-0.005 to 0.006)	0.873
Women (vs. men)	0.061 (-0.081 to 0.204)	0.400	0.017 (-0.135 to 0.171)	0.822
BMI, kg/m^2	**-0.027 (-0.049 to -0.004)**	**0.017**	**-0.029 (-0.052 to -0.007)**	**0.010**
Diabetes duration, year	0.061(-0.082 to 0.204)	0.404	0.136 (-0.017 to 0.289)	0.081
uACR, log unit	-0.019 (-0.138 to 0.099)	0.745	-0.102 (-0.241 to 0.036)	0.148
Angiotensin blockers use (yes vs. no)	-0.148 (-0.368 to 0.071)	0.185	-0.222 (-0.473 to 0.029)	0.083
FPG, mg/dL	**0.014 (0.003 to 0.024)**	**0.011**	**0.014 (0.004 to 0.025)**	**0.008**
Hemoglobin, g/dL	-0.002 (-0.045 to 0.041)	0.911	0.012 (-0.032 to 0.055)	0.604

Abbreviations: uNAG, urinary N-acetyl-β-D-glucosaminidase; BMI, body mass index; uACR, urinary albumin-to-creatinine ratio. In multivariate model, β coefficient of uNAG for G/A ratio was reported with adjustment for age, sex, BMI, duration of diabetes, log-uACR, angiotensin blockers use, FPG, and hemoglobin.

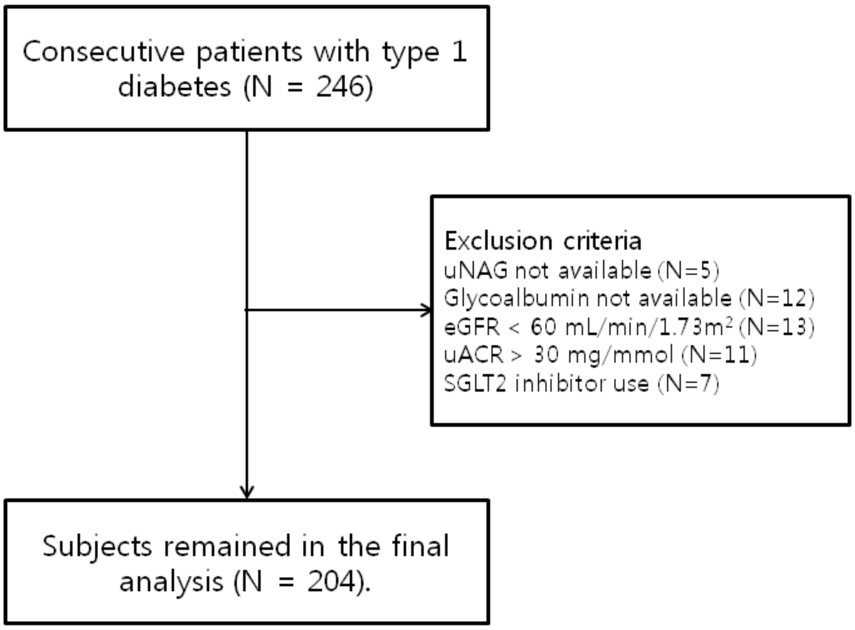

Supplementary Figure 1. Study flow diagram. Abbreviations: uNAG, urinary N-acetyl-β-D-glucosaminidase; uACR, urinary albumin-to-creatinine ratio; eGFR, estimated glomerular filtration rate; SGLT2, sodium-glucose co-transporter 2.

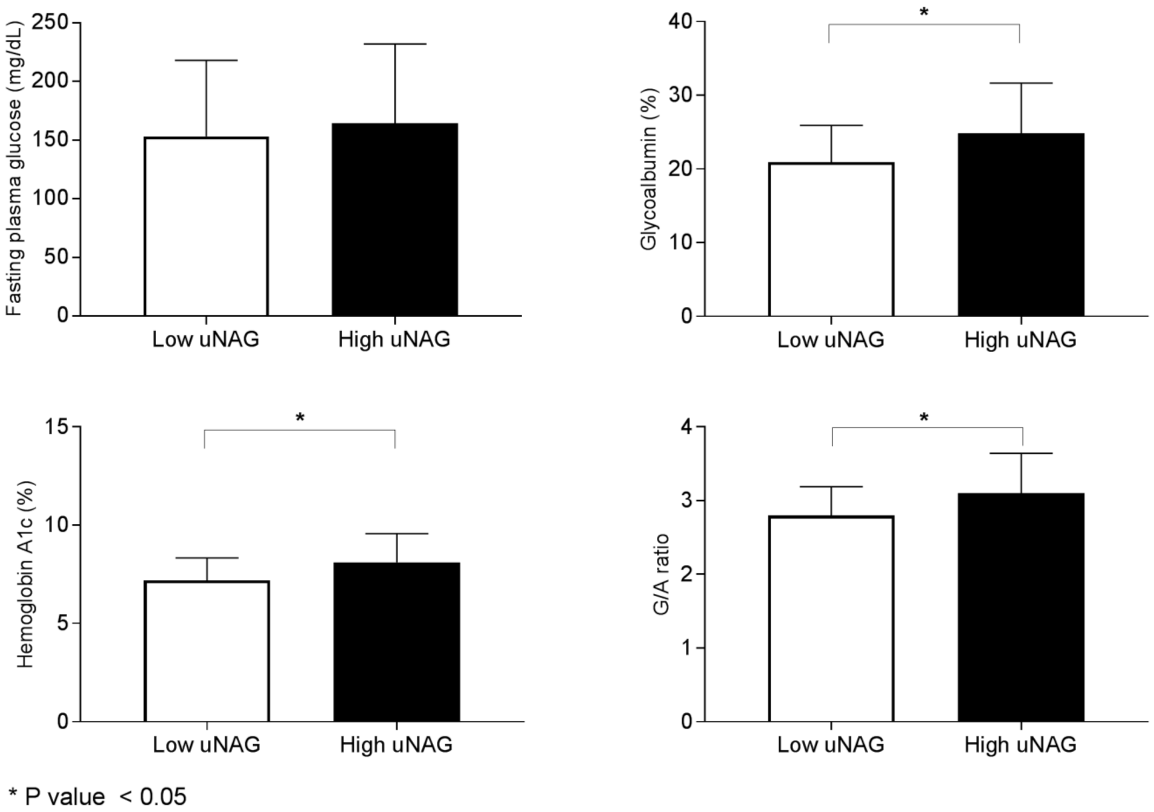

Supplementary Fig. 2. Comparison of glycemic parameters by uNAG level in a subset of subjects with normal to mildly increased albuminuria (A1, albumin-to-creatinine ratio (ACR) less than 30 mg/g creatinine). Low uNAG was defined as uNAG < 5.80 U/g creatinine, whereas high uNAG indicate uNAG ≥ 5.80 U/g creatinine. Abbreviations: uNAG, urinary N-acetyl-β-D-glucosaminidase; G/A ratio, glycoalbumin-to-hemoglobin A1c ratio.
